# Respiratory microbiome-host interaction on lung carcinogenesis, immunity, and immunotherapy

**DOI:** 10.3389/fimmu.2025.1676302

**Published:** 2025-11-28

**Authors:** Peng Wang, Cuihong Ge, Xinru Jing, Qijia Han, Mingzhu Wang, Minghua Huang, Zhiming Xiang

**Affiliations:** 1 Postgraduate Cultivation Base of Guangzhou University of Chinese Medicine, Panyu Central Hospital, Guangzhou, Guangdong, China; 2 Department of Radiology, The Affiliated Panyu Central Hospital, Guangzhou Medical University, Guangzhou, Guangdong, China; 3 School of Life Sciences, South China Normal University, Guangzhou, Guangdong, China; 4 Department of Gynecology, The Affiliated Panyu Central Hospital, Guangzhou Medical University, Guangzhou, Guangdong, China

**Keywords:** respiratory microbiome, non-small cell lung cancer, immunity, immunotherapy, antibiotics

## Abstract

The respiratory microbiome, as an integral component of the lung cancer microenvironment, exerts pivotal influences on tumorigenesis, immune homeostasis, and therapeutic response through intricate crosstalk with host immunity. Despite advancements, current limitations in lung cancer immunotherapy persist, including heterogeneous therapeutic responses, immune-related adverse events, and the lack of predictive biomarkers. These unmet clinical needs underscore the imperative to delineate the complex immune landscape of respiratory microbiome in lung cancer pathogenesis. This review systematically analyzes the hallmarks of respiratory dysbiosis (reduced α-diversity and enrichment of *Streptococcus* and *Veillonella*) and their associations with lung cancer staging, histological subtypes, and prognosis. We further elucidate how these microbial alterations influence tumor progression via metabolic-epigenetic-immune pathways. Additionally, we establish clinical correlations between microbiome signatures and both immune checkpoint inhibitor therapeutic efficacy/toxicity profiles, while examining the paradoxical effects of antibiotic exposure during immunotherapy. Emerging intervention strategies targeting the respiratory microbiome, such as aerosolized probiotics, engineered bacteria (e.g., *Escherichia coli*), and microbiota-derived nanomaterials, showcase potential in remodeling antitumor immunity and improving therapeutic outcomes. Our findings highlight the double-edged sword effect of the respiratory microbiota as biomarkers and therapeutic targets in lung cancer management, providing critical insights for clinical translation.

## 1 Introduction

Lung cancer represents the leading cause of cancer-related mortality worldwide, accounting for 18.7% of all deaths across all cancer types. Its pathogenesis is complex, involving genetic predisposition, environmental exposures, and microbiome dysbiosis (1). Due to the insidious nature of early-stage symptoms, approximately 60% of lung cancer patients are diagnosed at advanced stages (2). Immune checkpoint inhibitors (ICIs), which disrupt inhibitory pathways such as cytotoxic T lymphocyte-associated antigen 4 (CTLA-4), programmed cell death-1 (PD-1) protein, and its ligand PD-L1 to potentiate antitumor immunity, have become a cornerstone of treatment for advanced non-small cell lung cancer(NSCLC), substantially improving 5-year survival rates (3, 4). Nevertheless, three unresolved scientific issues hinder progress (1): Heterogeneous therapeutic responses limit durable clinical benefits to 20–50% of patients (5) (2); Immune-related adverse events (irAEs) affect nearly half of the treated individuals (incidence: 43%) (6) (3); Existing predictive biomarkers, including PD-L1 expression and tumor mutational burden, exhibit suboptimal specificity for identifying true responders (7). Therefore, advancing systematic biomarker identification and optimizing clinical therapeutic efficacy remain critical scientific challenges requiring breakthroughs in lung cancer immunotherapy research.

In recent years, the synergistic development of multi-omics technologies—including metagenomic sequencing, single-cell transcriptomics, and spatial transcriptomics—has overcome the spatiotemporal resolution limitations of traditional microbiome research. These advances have not only confirmed the existence of low-biomass dynamic microbial communities in the respiratory tract under physiological conditions but also revealed their dynamic interactions with the lung cancer immune microenvironment (8). Clinically, lung cancer patients exhibit marked shifts in commensal microbial diversity and taxonomic abundance, where relative abundance of signature taxa (e.g., *Streptococcus*) correlates with disease trajectory and survival outcomes (9). Emerging preclinical evidence highlights beneficial microbiota transplantation and metabolic intervention (e.g., short-chain fatty acids and tryptophan derivatives) as potent adjuvants to PD-1/PD-L1 blockade, achieving near-abrogation of tumor growth in murine models (10, 11). Notably, microbiome dysbiosis may conversely exacerbate tumor progression and diminish therapeutic responses, particularly to immunotherapy (12). Mechanistically, commensal microbiota can influence immunotherapy efficacy by modulating immune cell activity and remodeling the tumor immune microenvironment (TIME) (including fostering an immunosuppressive environment or enhancing immune surveillance) (13). Consequently, restoring microbial homeostasis through probiotics, prebiotics, and postbiotics has emerged as a novel research paradigm in comprehensive cancer care, providing critical insights into the heterogeneity of immunotherapy outcomes and guiding personalized treatment strategies (14).

In this review, we systematically explore the multidimensional interaction network between the respiratory microbiome and lung cancer pathogenesis and immunotherapy efficacy, with a focus on the molecular mechanisms underlying microbiome-mediated modulation of immunotherapy outcomes and its translational clinical value. Finally, through critical evaluation of current preclinical limitations, we propose targeted future research directions and optimization strategies for leveraging the respiratory microbiome in precision lung cancer therapy.

## 2 Relationship between respiratory microbiome and clinical features of lung cancer

The pathogenesis of lung cancer constitutes a multifactorial, multistage process wherein microbiome-tumor microenvironment interactions play a critical regulatory role. Emerging evidence highlights that the compositional and functional heterogeneity of microbial communities across distinct anatomical regions of the respiratory tract may hold unique biological significance in lung carcinogenesis (15). Salivary microbiota is postulated as a primary source for pulmonary microbial colonization (16). The dysbiosis of salivary microbiota, which is characterized by diminished alpha diversity(95% CI 0.84-0.96) and *Streptococcus*-dominant enrichment(95% CI 1.06-1.22), correlates with heightened malignancy risk (17). Notably, lung cancer patients demonstrate markedly lower salivary microbial diversity and richness compared to healthy controls, while specific microbial taxa such as *Capnocytophaga*, *Veillonella*, *Sphingomonas*, and *Blastomonas* display significant enrichment (8, 18), implicating salivary microbiota as potential biomarkers for lung cancer. Sputum-derived microbial profiles provide more direct insights into lower respiratory tract ecology. Leng et al. (19) revealed that the abundance of *Acidovorax* and *Veillonella* was significantly increased in sputum of NSCLC patients by Droplet digital PCR, underscoring their biomarker potential for early detection and tumor classification. Metagenomic signatures further implicate *Streptococcus viridans* overabundance in sputum as a progression-associated indicator of lung cancer (20). Bronchoalveolar lavage fluid (BALF), the gold standard for detecting the lung microbiome in clinical settings, enables precise characterization of the peritumoral microenvironment. BALF microbial alterations not only associate closely with lung cancer development, progression, and histological subtypes (21), but also offer novel approaches for early detection (22). Multiple studies demonstrate reduced species diversity and richness in BALF microbiota from lung cancer patients compared to healthy controls, dominated by Bacillota, Pseudomonadota, Bacteroidota, Actinomycetota, and Fusobacteriota, with additional enrichment of Cyanobacteriota, Saccharibacteria*, and* genera including *Prevotella, Streptococcus, Veillonella, Neisseria, Haemophilus, Clostridium*, and *Actinobacillus (*23, 24). Wang et al. report diminished microbial diversity in both saliva and BALF samples from lung cancer patients, identifying *Treponema* and *Filifactor* in BALF as potential diagnostic biomarkers (25). Furthermore, significant differences in BALF microbiota exist between lung squamous cell carcinoma(LUSC) and adenocarcinoma, with Pseudomonadota enrichment in LUSC patients—particularly among males and heavy smokers—potentially linked to tumor invasiveness and metastatic potential (26).

Lung cancer exhibits significant spatial heterogeneity in microbial composition between intratumoral and peritumoral tissues. Bingula et al. (27) characterized unique microbiome signatures in saliva, BALF, peritumoral lung tissues, and tumor tissues, which showed associations with tumor localization, histological subtype, and immune activation. Notably, compared to peritumoral tissue microbiomes, the intratumoral microbiome is least influenced by anatomical location. Furthermore, Peters et al. (28) revealed that the microbial diversity in peritumoral tissues is significantly associated with the prognosis of NSCLC patients. Subsequent investigations have delineated specific relationships between intratumoral microbial diversity, abundance shifts of particular taxa, and oncogenesis. For instance, Yu et al. (29) reported increased *Thermus* abundance in advanced-stage patients and *Legionella* enrichment in metastatic cases. Li et al. (30) identified marked microbiome differences between malignant and non-malignant lung tissues in advanced NSCLC, particularly enrichment of Pseudomonadota (predominantly *Acinetobacter and Acidovorax*), Bacillota, and Actinomycetota. Smoking-related lung cancer tissues showed a correlation between *Acidovorax* enrichment and *TP53* mutations (31), while Apopa et al. (32) detected Cyanobacteriota prevalence in lung adenocarcinomas with microcystin levels linked to PARP1 overexpression. Collectively, these findings support the existence of tumor-associated microbiome patterns in lung carcinogenesis and progression. Prognostically, intratumoral microbiota features show significant associations with recurrence and metastasis. Zhou et al. (33) demonstrated the predictive value of intratumoral microbiota for recurrence/metastasis risk in LUSC, with microbial risk scores correlating with survival outcomes. Patnaik et al. (34) established associations between preoperative lower respiratory tract microbiota and early NSCLC recurrence. Deng et al. (35) developed a translational prognostic model integrating 18 microbial taxa with a 19-gene glycolysis-lactate signature. Ma et al. (36) identified butyrate-producing bacteria (e.g., *Roseburia*) enrichment in recurrent cases, mechanistically linking butyrate-mediated HDAC2 inhibition to H3K27 hyperacetylation, upregulation of H19 expression, and M2 macrophage polarization-driven metastasis. These advancements underscore the diagnostic and prognostic potential of respiratory microbiota in lung cancer management (**Table 1**).

**Table 1:** Studies investigating the composition of the microbiome in different sample types obtained from patients with NSCLC.

Sample type	Author year [ref]	Sample size	Analytic method	Microbiome findings	Major findings
Saliva, BAL, nasal, gastric	Bassis et al., 2015 (16)	28 patients	16S rRNA sequencing, qPCR	The lungs selectively eliminate *Prevotella* bacteria from the upper airways.	Microaspiration likely common; lung microbiome overlaps with oral but not nasal microbiota
Saliva	Vogtmann et al., 2022 (17)	1306 patients	16S rRNA sequencing	Higher alpha diversity associated with lower lung cancer risk; *Streptococcus* implicated	Oral microbiota linked to lung cancer risk; associations vary by smoking history and histologic subtype
Saliva	Yan et al., 2015 (18)	86 patients	16S rRNA sequencing	*Capnocytophaga, Veillonella*, and *Neisseria* were elevated in lung cancer	*Capnocytophaga* and *Veillonella* as biomarkers for SCC and ADC
Sputum	Leng et al., 2021 (19)	107 patients	Droplet digital PCR	*Acidovorax, Veillonella*, and *Capnocytophaga* as diagnostic biomarkers for NSCLC	Sputum microbiome might provide noninvasive biomarkers for the early detection and classification of NSCLC.
Sputum	Cameron et al., 2017 (20)	10 patients	Metagenomic sequencing	*Streptococcus viridans* and *Granulicatella adiacens* as potential biomarkers for Lung cancer	*G. adiacens* abundance could be related to Lung cancer stage.
BALF, sputum	Huang et al., 2019 (21)	40 BALF, 52 sputum	16S rRNA sequencing	Pseudomonadota higher in BALF; *Veillonella, Megasphaera, Capnocytophaga* differential between SCC and ADC	BALF better reflects lung cancer microbiome; microbial differences between metastatic states and histologic types
BALF	Marshall et al., 2022 (22)	72 patients	16S rRNA sequencing	*Veillonella, Streptococcus, Prevotella*, and *Paenibacillus*	Microbiome changes precede clinical lung cancer diagnosis, offering potential for early detection
BALF	Jin et al., 2019 (23)	150 discovery, 85 validation	Metagenomics analysis	*Bradyrhizobium japonicum* unique to cancer; reduced richness in lung cancer	The lower respiratory tract microbiome richness is diminished in lung cancer patients compared with that in healthy subjects.
BALF	Liu et al., 2018 (24)	24 patients, 18 controls	16S rRNA sequencing	*Streptococcus* more abundant in cancer; alpha diversity decreased in cancer	Lung cancer-associated microbiota profile distinct from healthy controls; *Streptococcus* as a potential biomarker
Saliva, BALF	Wang et al., 2019 (25)	51 patients, 15 controls	16S rRNA sequencing	*Treponema, Filifactor* identified as potential biomarkers; reduced diversity in cancer	Lung cancer patients have a distinct, less diverse microbial community compared to healthy individuals. Specific bacterial groups may be linked to lung cancer, with the exact species varying by sampling location and cancer type.
BALF	Gomes et al., 2019 (26)	23 patients	16S rRNA sequencing	Lung cancer microbiota is enriched in Pseudomonadota and more diverse in SCC than ADC; specific taxa linked to survival	Microbial diversity and composition correlate with lung cancer subtype and patient survival
Saliva, BALF, lung tissue	Bingula et al., 2020 (27)	28 patients	16S rRNA sequencing	Pseudomonadota dominated tissue samples, while Bacillota was more abundant in BALF and saliva.	Microbiome composition varies by sample type, suggesting BAL may not fully represent lung microbiome
Lung tissue	Peters et al., 2019 (28)	19 patients	16S rRNA sequencing	Higher abundance of family *Koribacteraceae, Bacteroidaceae, Lachnospiraceae*, and *Ruminococcaceae* in normal tissue linked to survival	Normal lung microbiota may influence lung cancer prognosis
Lung tissue	Yu et al., 2016 (29)	165 patients	16S rRNA sequencing	*Thermus* is more abundant in tissue from advanced-stage patients, while *Legionella* is higher in patients who develop metastases.	Smoking and environmental exposures significantly impact lung microbiome diversity
Lung tissue	Li et al., 2023 (30)	67 patients	Metagenomic shotgun	Enriched Pseudomonadota, Bacillota, and Actinomycetota in NSCLC tumors	Lower microbiota diversity in tumors; upregulation of proinflammatory cytokines
Lung tissue	Greathouse et al., 2018 (31)	143 patients	16S rRNA sequencing	*Acidovorax* enriched in TP53-mutated squamous cell carcinoma	TP53 mutations interact with microbiome in SCC
Lung tissue	Apopa et al., 2018 (32)	40 patients	16S rRNA sequencing	Cyanobacteriota enriched in ADC; PARP1 up-regulated in microcystin-exposed cells	Microcystin from Cyanobacteriota promotes lung carcinogenesis
Lung tissue	Zhou et al., 2023 (33)	Multi-omics data	16S rRNA sequencing, RNA sequencing	In SCC, *Shigella, Staphylococcaceae, Staphylococcus, Pseudogulbenkiania*, and *Chromobacteriaceae* were enriched in RM, while *Leuconostocaceae, Acidovorax, Shewanellaceae, Shewanella*, and *Comamonadaceae* were enriched in the non-RM group	The microbial diversity of SCC recurrence and metastasis groups was low, and a prediction model was constructed (AUC = 0.81), predicting risk was significantly associated with patient survival
Lung tissues, BALF and saliva	Patnaik et al., 2021 (34)	47 patients	16S rRNA sequencing	*Staphylococcus, Bacillus, Anaerobacillu*s differ between recurrent and non-recurrent cases	Presurgery composition of lower airway microbiome may be associated with recurrence of early NSCLC.
Lung tissue	Deng et al., 2023 (35)	TCGA-LUAD dataset	16S rRNA sequencing, RNA sequencing	18-microbe prognostic score; glycolysis-lactate signature predicts prognosis	Microbiome and glycolysis-lactate pathways are linked to ADC prognosis and immunotherapy response
Lung tissue	Ma et al., 2024 (36)	150 discovery, 85 validation	16S rRNA sequencing, RNA sequencing	Butyrate-producing bacteria (*Roseburia*) enriched in recurrent cases	Butyrate promotes metastasis via H19 expression; microbial signature predicts recurrence

ADC, adenocarcinoma; BALF, Bronchoalveolar Lavage Fluid; NSCLC, non-small cell lung cancer; RM, recurrence and metastasis; SCC, squamous cell carcinoma.

## 3 Possible mechanisms of the microbiome on lung cancer pathogenesis

The dual role of the respiratory microbiome in lung cancer progression: Maintaining immune homeostasis versus driving inflammation during dysbiosis. As a pivotal regulator of the TIME, the respiratory microbiota exerts significant influence on pulmonary immune equilibrium and oncogenesis through multidimensional modulation of innate and adaptive immune networks (37). These biological effects appear contingent upon the immunogenicity and colonization patterns of specific microbial species (38). Within the adaptive immune system, a sophisticated regulatory network emerges through the Treg/Th17 cell balance. Research reveals intricate connections between neonatal lung microbial colonization, reduced airway hyperresponsiveness, and Treg cell subset (39). Healthy lower airways exhibit a characteristic microbial profile dominated by stable colonization of oropharyngeal commensals, including *Prevotella, Veillonella*, and *Streptococcus (*40). This colonization primarily occurs through microaspiration pathways, establishing a dynamic equilibrium with the respiratory epithelium (41). These commensal communities demonstrate significant interactions with Th17-mediated mucosal immunity, crucially modulating the balance between pulmonary immune surveillance and pathological inflammatory responses (42). The pulmonary innate immune defense comprises alveolar macrophages and γδT cell populations. Through BALF multi-omics analysis, Zheng’s team revealed that altered lung microbiota in NSCLC patients associates with suppressed tumor growth via M2 macrophage reduction and enhanced CD3^+^/CD8^+^T cell infiltration (43). Mechanistic investigations further demonstrate that lung commensals sustain γδT cell-mediated antitumor responses through alveolar macrophage regulation of CCL24 chemokine production (44). Crucially, pulmonary microbiota orchestrates tumor immune surveillance through γδT17-dependent mechanisms, playing indispensable roles in immune cell regulation, barrier maintenance, and host antitumor immunity coordination (45, 46). 

**Figure 1:**
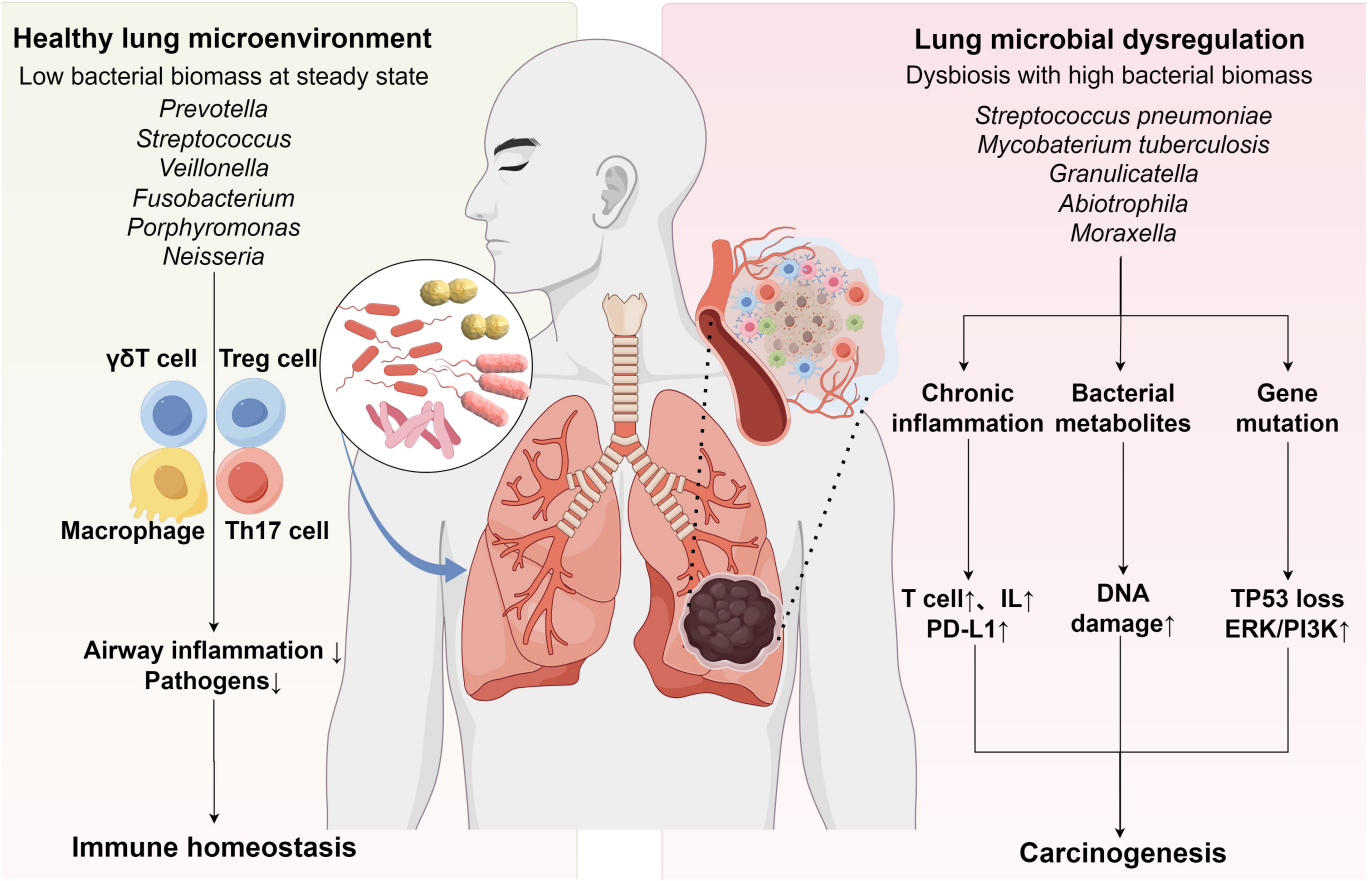
Interactions among the Respiratory Microbiota, Immune Homeostasis, and Lung Cancer. Under physiological conditions, commensal microbiota establishes multi-tiered immunoregulatory mechanisms by dynamically coordinating innate immunity (via Treg/Th17 balance regulation) and adaptive immunity (through alveolar macrophage polarization and γδT cell functional differentiation). This synergistic interaction prevents excessive inflammation while maintaining pulmonary immune homeostasis (Left panel). Respiratory dysbiosis may promote lung carcinogenesis through three interconnected mechanisms: chronic inflammation triggered by immune dysregulation, microbiota-derived metabolite-mediated epigenetic modifications, and host-microbe interaction-associated genetic mutations coupled with aberrant activation of oncogenic signaling pathways (Right panel).

Emerging evidence establishes that respiratory microbiome dysbiosis promotes lung carcinogenesis through multifactorial immunomodulatory pathways, which can be systematically categorized into three principal biological mechanisms (1): chronic inflammation secondary to immune homeostasis disruption (2), epigenetic modulation via microbial metabolites, and (3) genetic mutation/signaling pathway activation through host-microbe interactions (47) (**Figure 1**). At the immunomodulatory level, pulmonary dysbiosis directly drives tumorigenesis by inducing Th17/γδ T cell-mediated inflammatory responses (48). Clinical studies have demonstrated an association between pulmonary dysbiosis in NSCLC patients and Th17-mediated pulmonary inflammation, where IL-17 secretion by these cells perpetuates chronic inflammation and accelerates malignant progression (49). Preclinical investigations further reveal that lung commensal bacteria activate γδ T cells to initiate inflammation linked to adenocarcinoma. Notably, germ-free or antibiotic-treated mice exhibit significant protection against Kras mutation- and p53 deletion-driven lung carcinogenesis (38), suggesting γδ T cell hyperactivation as a critical microbiome-dependent mechanism in inflammation-driven malignancy. Microbial metabolites exhibit dual regulatory roles in epigenetic modulation (50). Reduced microbial diversity and increased *Streptococcus* abundance characterize the lower respiratory tract microbiome in lung cancer patients. *Streptococcus pneumoniae*-derived pneumolysin and pyruvate oxidase may promote carcinogenesis by disrupting host cell metabolism and apoptosis (24, 51). Cyanobacteriota-derived microcystins correlate with reduced CD36 and elevated PARP1 levels, suggesting therapeutic potential through microcystin transport inhibition or PARP1 targeting (32). Conversely, beneficial metabolites like butyrate (a short-chain fatty acid) and indole-3-aldehyde (I3A) demonstrate anticancer properties. Butyrate modulates miRNA expression in NSCLC A549 cells to suppress proliferation (52) and inhibits HDAC3 to drive monocyte-to-macrophage differentiation, thereby reducing inflammatory mediators and enhancing antimicrobial activity (53). The interaction between probiotic-derived I3A and tumor-infiltrating CD8^+^ T cells further enhances antitumor immunity (54). Aspects of genetic mutations and signaling pathways, microbiome-smoking interactions correlate with TP53 mutations in LUSC, particularly the enrichment of polycyclic aromatic hydrocarbon-degrading genera (*Acidovorax, Massilia*) in smokers’ tumor microbiota (31). Lower airway enrichment of *Streptococcus and Veillonella* in lung cancer patients associates with ERK/PI3K pathway activation, potentially fostering tumor progression (55). Intriguingly, these bidirectional regulatory mechanisms may extend to immunotherapy responses, though mechanistic details remain to be elucidated. 

## 4 Microbiota and lung cancer immunotherapy

### 4.1 Microbiota and efficacy of immunotherapy in lung cancer

The microbiome exerts dual regulatory effects on immunotherapy efficacy through distinct immunomodulatory pathways mediated by respiratory and gut microbiota (56). First, respiratory microbiota (particularly the abundance of specific bacteria) directly interact with the tumor immune microenvironment through local immunomodulation (57). For instance, Jang et al. (58) demonstrated that elevated *Veillonella dispar* abundance positively correlates with PD-L1 upregulation and enhanced immunotherapy responsiveness, whereas Gammapseudomonadota predominance associates with PD-L1 suppression and unfavorable prognosis. Furthermore, Zapata et al. (59) identified *Gemella* abundance in respiratory microbiota as a potential predictor of ICI resistance, while *Lachnoanaerobaculum* abundance shows potential as a biomarker for favorable ICI response. Notably, intratumoral microbial heterogeneity in lung cancer, particularly *Fusobacterium* enrichment, has been linked to immunotherapy resistance (60). Second, gut microbiota mediates systemic immunomodulation through the gut-lung axis, significantly impacting treatment outcomes (61). Substantial evidence reveals that ICI-responsive NSCLC patients typically exhibit enriched populations of *Bifidobacterium (*9), *Akkermansia muciniphila*(AKK) *(*62), *Bacteroides (*63), and *Ruminococcus* (64) in gut microbiota, with these microbial profiles correlating with improved PFS following ICI therapy (65).

With the widespread application of ICIs in lung cancer treatment, approximately 40% of patients experience irAEs affecting multiple organ systems, including the skin, gastrointestinal tract, and cardiopulmonary systems (66). Notably, irAEs exhibit a complex double-edged sword effect of clinical efficacy: while their occurrence correlates with improved patient survival (6), severe immune toxicities induced by ICIs may lead to treatment discontinuation or life-threatening complications (67). Consequently, identifying early predictive biomarkers for severe irAEs has become a priority in ICI patient management (68). In recent years, the microbiome has gained attention as a potential biomarker for predicting ICI efficacy and toxicity. The MITRE trial pioneered the evaluation of microbiome signatures as biomarkers to assess treatment response and toxicity in cancer patients receiving ICIs, aiming to elucidate dynamic associations between microbial features and clinical outcomes (69). Chau et al. (70) investigated correlations among nasal, oral, and gut microbiomes, treatment response, and irAEs in lung cancer patients undergoing ICI therapy, revealing significantly reduced gut microbiome alpha-diversity in these patients, which was strongly associated with both therapeutic response and irAEs. The Liu team further demonstrated that gut microbiota alterations in anti-PD-1-treated lung cancer patients were linked to immune-related diarrhea (71). Regarding microbial intervention strategies, clinical studies indicate that NSCLC patients receiving probiotics during immunotherapy exhibit significantly prolonged progression-free survival (PFS) and overall survival (OS), correlating with favorable clinical outcomes (72). Shaikh et al. (73) proposed that modulating the microbiome during treatment or applying microbiota transplantation might optimize therapeutic effects and mitigate irAEs. Mechanistic studies suggest that *Clostridium butyricum* supplementation enhances survival rates and ICI responsiveness in lung cancer patients (74), while *AKK* potentiates the anti-tumor efficacy of IL-2 immunotherapy (75). Additionally, *Lactobacillus rhamnosus Probio-M9* has been shown to reverse antibiotic-induced dysbiosis and improve ICI efficacy (76). Chen et al. (77) reported that the postbiotic JK5G alleviates irAEs in advanced NSCLC patients receiving ICIs, demonstrating potential to enhance treatment outcomes while reducing adverse events. Collectively, these findings position the lung cancer microbiome as a promising diagnostic and predictive biomarker platform, potentially enabling patient stratification and treatment optimization.

### 4.2 Influence of antibiotics on immunotherapy efficacy in patients with lung cancer

The impact of antibiotic use on ICI efficacy in NSCLC has garnered significant attention in recent years. While current research generally suggests that antibiotics may compromise immunotherapy outcomes by disrupting commensal microbiota, current findings demonstrate notable heterogeneity. Substantial clinical evidence indicates that antibiotic exposure correlates with adverse prognostic outcomes and reduced ICI efficacy, with dose-dependent effects (59). Notably, Derosa et al. (78) documented a 5.5-month reduction in median PFS and 14.3-month decrease in OS among NSCLC patients receiving β-lactams or quinolones versus controls (*P* < 0.05). These findings gain support from a comprehensive meta-analysis by Abdelhamid et al. (79) (19 studies, n=2,932), demonstrating significant associations between antibiotic use and inferior survival outcomes (PFS HR = 1.64, OS HR = 1.67) in ICI-treated cohorts. Notably, antibiotic administration within ±60 days of treatment initiation correlates with diminished survival outcomes (80). Castello et al. (81) identified correlations between antibiotic exposure, elevated tumor metabolic burden, and accelerated disease progression. Beyond therapeutic efficacy, antibiotics may exacerbate irAEs. Jing et al. (82) reported a 1.39-fold higher risk of irAEs (95% CI 1.21-1.59) in antibiotic-exposed NSCLC patients, particularly evident in those receiving anti-PD-1/PD-L1 therapies. Preclinical models reinforce these clinical patterns: Antibiotic-treated mice exhibited enhanced Lewis lung carcinoma progression with reduced survival and increased pulmonary tumor burden (46). Routy et al. (83) revealed that oral antibiotics impair ICI efficacy, while fecal microbiota transplantation from ICI-responsive patients (enriched with AKK) restored anti-tumor responses to PD-1 blockade in germ-free or antibiotic-pretreated mice. Furthermore, Tan et al. (84) observed that although antibiotics aggravated ICI-associated colitis in murine models, specific probiotic supplementation mitigated these effects. Recent mechanistic insights highlight the microbiota-immune axis as a critical therapeutic determinant. An intact gut microbiome facilitates optimal treatment responses by modulating myeloid-derived cell functions within the tumor microenvironment (85). Conversely, antibiotic-induced dysbiosis promotes the migration of immunosuppressive intestinal Treg/Th17 cells to tumors via the MAdCAM-1-α4β7 axis, establishing an immunoinhibitory microenvironment that compromises PD-1 blockade efficacy (86).

However, other studies have failed to establish statistically significant associations between antibiotic exposure and survival outcomes in lung cancer patients. While Hakozaki et al. (87) reported a potential association between antibiotic use and reduced PFS in NSCLC patients through univariate analysis (*P* = 0.04), this finding lost statistical significance in multivariate models. Similarly, Nyein et al. (88) identified a non-significant trend toward worse OS in antibiotic-exposed NSCLC patients receiving immunotherapy (HR = 1.35, *P* = 0.145). In epidemiological investigations, Zhang et al. (89) observed an attenuated association between frequent antibiotic prescriptions (≥10 courses) and lung cancer risk after covariate adjustment (RR = 2.52 vs 1.31), concluding insufficient evidence for antibiotic-induced carcinogenesis. Preclinical findings by Noci et al. (90) revealed that aerosolized antibiotic treatment reduced pulmonary bacterial load in mice, decreased Treg cell populations, and enhanced T-cell/NK cell activity, thereby significantly inhibiting B16 melanoma lung metastasis and potentiating antitumor immunity. The divergent outcomes between aerosolized and oral antibiotic administration may stem from their distinct microbial targets: Systemic oral antibiotics primarily disrupt gut microbiota, potentially impairing the gut-lung axis and systemic antitumor immunity, whereas localized aerosol therapy selectively modulates respiratory microbiota to reshape the immunosuppressive pulmonary microenvironment. These findings underscore the need for judicious consideration of infection management strategies and anatomical site-specific microbiome modulation in lung cancer therapeutics.

## 5 The translational medical value of respiratory microbiota modulation in lung cancer immunotherapy

### 5.1 Aerosolized probiotics

Targeted drug delivery strategies leveraging respiratory tract anatomy have revolutionized lung cancer therapy. Aerosolized inhalation systems, distinguished by their favorable pharmacokinetic profiles, enable non-invasive, site-specific delivery of therapeutics across the air-blood barrier. This approach enhances localized drug deposition in tumor microenvironments while mitigating systemic toxicity (91). Notably, the Le Noci team demonstrated that aerosolized immunostimulants enable repeatable dosing in metastatic lung cancer patients, curtailing M2 macrophage polarization and boosting anti-tumor effects (92). Subsequent work established that aerosolized antibiotics/probiotics remodel pulmonary niches to foster anti-metastatic immunity (90). Further investigations showed that aerosolized live or inactivated *Lactobacillus rhamnosus* impeded murine lung tumorigenesis, marked by decreased tumor burden, reduced Treg infiltration, and elevated IgA titers (93). Aerosolized probiotic formulations may reverse immunosuppressive pulmonary microenvironments through dual mechanisms of microbial community restoration and immune tolerance modulation, thereby enhancing antitumor efficacy in lung cancer (94). Supporting evidence includes Zheng et al.’s findings (43) showing that inhalation of NSCLC patients’ lung microbiota induces significant compositional shifts in murine pulmonary microbiomes (with *Pasteurella* replacing *Delftia* as the dominant genus), subsequently inhibiting lung cancer cell proliferation. Youn et al. (95) revealed that the intranasal administration of viable *Lactobacillus* conferred stronger protection against murine influenza infection than oral delivery, with live bacteria exhibiting superior efficacy to inactivated counterparts.

### 5.2 Engineered bacteria

Engineered bacterial systems utilizing synthetic biology have emerged as a promising frontier in lung cancer therapeutics, particularly excelling in targeted drug delivery and immunomodulation (96). Current research bifurcates into two primary strategies: engineering bacterial outer membrane vesicles (OMVs) and developing programmable live bacteria. Distinct from conventional treatments, this technology platform combines three fundamental advantages: 1) Coordinated activation of innate and adaptive immunity to enhance therapeutic efficacy while reducing off-target toxicity; 2) Tumor-specific colonization through bacteria’s inherent immunogenicity; 3) Implementation of tumor microenvironment-responsive drug release through genetic engineering (97). Notably, engineered OMVs demonstrate enhanced immunotherapeutic specificity (98). Chen et al. (99) innovatively integrated OMV-coated drug-loaded polymeric micelles, where OMVs activate immune responses while micellar components simultaneously execute chemotherapy and immune sensitization of cancer cells to cytotoxic T lymphocytes. Kuerban et al. (100) developed attenuated *Klebsiella pneumoniae*-derived OMVs loaded with doxorubicin (DOX-OMV), demonstrating superior cell targeting and cytotoxicity in A549 lung adenocarcinoma models, coupled with potent tumor suppression *in vivo*. Parallel advancements include Gurbatri et al. ‘s probiotic system for localized PD-L1/CTLA-4 nanobody delivery (101) and Chowdhury et al.’s tumor microenvironment-responsive *E. coli* strain releasing CD47-blocking nanobodies, which collectively enhance T cell infiltration, induce tumor regression, and inhibit metastasis in preclinical models (102).

Specific engineered bacterial strains have demonstrated dual functionality as both delivery vectors for antitumor drugs and active modulators of tumor immunity. These strains stimulate the host immune system, enhance the presentation of tumor-associated antigens, and amplify effector T cell activity to achieve therapeutic effects (102). For example, engineered commensal microbes show promise in preventing cancer initiation and inducing regression in colorectal cancer (CRC) (103). Among these, *Escherichia coli Nissle 1917 (EcN)* is the most extensively studied engineered strain. Leveraging its intrinsic tumor-colonizing capability and well-established safety profile in humans, *EcN* has become a premier platform for synthetic biology applications (104). The Canale research team developed metabolically engineered *EcN* strains that continuously convert ammonia to L-arginine within the tumor microenvironment. This metabolic reprogramming markedly improved mitochondrial function and survival of CD8+ T cells, enhanced tumor infiltration depth and cytotoxic activity of effector T cells, and ultimately elevated therapeutic response rates to ICIs (105). To optimize *EcN*’s bioavailability, Xie et al. engineered a prebiotic-based “barrier” system that not only increased *EcN*’s survival in simulated gastric acid but also extended its intestinal retention time. Mechanistic investigations revealed that this prebiotic-*EcN* synergy reshapes gut microbiota composition and stimulates the production of SCFAs, particularly butyrate, offering an innovative strategy for managing inflammation-associated CRC (106). Further studies combining probiotics with prebiotics demonstrated that orally administered prebiotic-coated probiotic spores (spores-dex) modulate the gut microbiome, enrich SCFA-producing bacteria (e.g., *Eubacterium* and *Roseburia*), and significantly boost overall microbial diversity. Notably, these spores exhibited specific enrichment within CRC cells, where they locally produced anticancer SCFAs, effectively suppressing tumor growth (107).

### 5.3 Microbiota-derived nanomaterials

**Figure 2:**
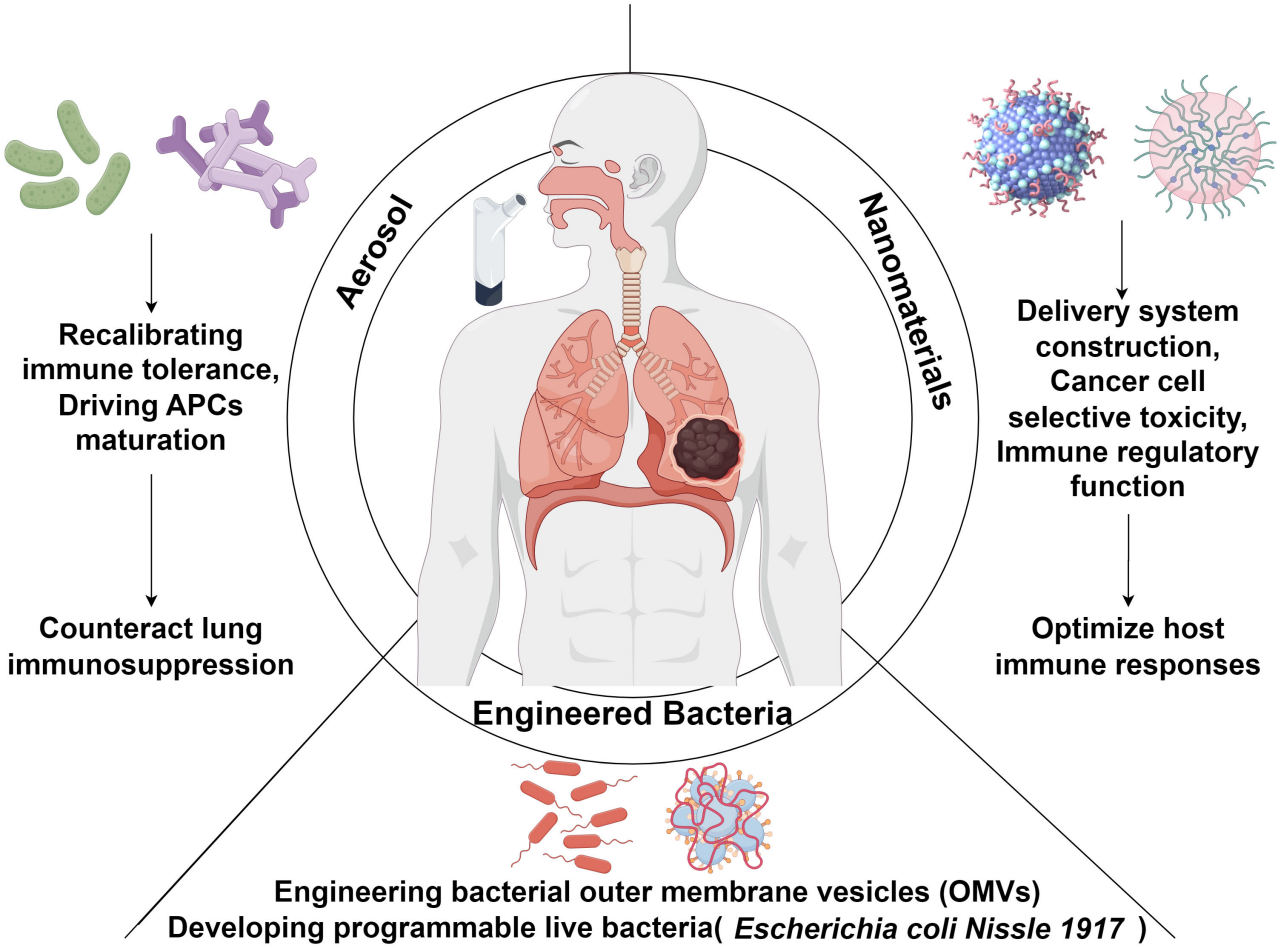
Schematic overview of probiotic-based strategies for modulating host immunity in lung cancer immunotherapy. Probiotic interventions, delivered via aerosolization, nanomaterial platforms, or bacterial engineering, offer novel avenues for enhancing antitumor immune responses in lung cancer. Aerosol delivery enables localized immune modulation by recalibrating immune tolerance and promoting the maturation of antigen-presenting cells (APCs), thereby mitigating the immunosuppressive tumor microenvironment within the lung. Nanomaterials can be tailored for targeted delivery, tumor-selective cytotoxicity, and immune-regulatory functions to potentiate host antitumor immunity. Advances in synthetic biology have enabled the engineering of bacterial outer membrane vesicles (OMVs) and programmable live microorganisms, such as Escherichia coli Nissle 1917 (EcN), further augmenting the efficacy and precision of lung cancer immunotherapeutic strategies.

Advances in nanotechnology have positioned probiotic-derived nanomaterials as promising agents for personalized medicine. In drug delivery systems, these materials enable precision-targeted tumor therapies. For instance, Li et al. (108) employed the anaerobic probiotic *Bifidobacterium infantis* as a pre-implanted carrier to recruit bacteria, achieving localized enrichment of nano-drug missiles in hypoxic tumor regions of lung cancer. This approach enhances probiotic stability and bioavailability while amplifying therapeutic outcomes and minimizing adverse effects (109). In selective anti-tumor research, metabolite-driven nanosynthesis techniques offer distinct advantages. The Repotente group engineered gold nanoparticles using *Lactobacillus acidophilus* metabolites, which selectively targeted breast cancer MCF7 and lung cancer A549 cells (IC50: 0.075 mM and 0.07 mM, respectively) *in vitro* but remained nontoxic to normal cells and myoblasts (110). Shehata et al. (111) synthesized exopolysaccharide-coated selenium nanoparticles, demonstrating their antioxidant and anti-lung cancer potential. However, the IC50 against A549 cells (5.324 µg/mL) highlighted efficacy disparities across nanomaterial systems. Nanomaterials can also enhance the host immune response by modulating the microbiome (112). Zheng et al. (113) further showed that silver nanoparticle-embedded mucoadhesive hydrogels, by modulating oral microbiota (*Peptostreptococcus*), potentiated PD-1 blockade efficacy in a mouse model of oral squamous cell carcinoma (**Figure 2**).

## 6 Conclusion

This review synthesizes current evidence on the interplay between the pulmonary microbiome and lung carcinogenesis, highlighting its therapeutic relevance in immunotherapy. Nevertheless, existing research exhibits critical limitations (1): Predominantly cross-sectional designs restrict insights into microbial dynamic evolution (2); Methodological variability in sampling sites (e.g., sputum, BALF, tissue biopsies) and sequencing protocols compromises reproducibility and cross-study comparability (3); While correlative evidence underscores associations between lung microbiota composition/diversity and cancer outcomes, causative mechanisms remain poorly characterized (4); Greater attention must be paid to the interaction between airway microbiota and the host, as well as the potentially distinct effects and mechanisms of antibiotics in the gut versus the lungs; Moving forward, multidisciplinary approaches integrating single-cell spatial transcriptomics, metabolomics, and multi-omics are imperative to decode the microbiome-tumor-immune axis. Rigorous multi-center trials are needed to validate microbial biomarkers for clinical translation, alongside innovative therapies to enhance efficacy and minimize toxicity (46). Additionally, the safety and ethical implications of microbiome modulation demand thorough consideration to ensure clinical feasibility and safety.
